# Sex-biased gene expression and sequence conservation in Atlantic and Pacific salmon lice (*Lepeophtheirus salmonis*)

**DOI:** 10.1186/s12864-016-2835-7

**Published:** 2016-07-04

**Authors:** Jordan D. Poley, Ben J. G. Sutherland, Simon R. M. Jones, Ben F. Koop, Mark D. Fast

**Affiliations:** Department of Pathology & Microbiology, Atlantic Veterinary College, University of Prince Edward Island, 550 University Ave, Charlottetown, PE C1A 4P3 Canada; Department of Biology, Centre for Biomedical Research, University of Victoria, 3800 Finnerty Rd, Victoria, BC V8W 3 N5 Canada; Pacific Biological Station, 3190 Hammond Bay Road, Nanaimo, BC V9T 6 N7 Canada; Present address: Département de biologie, Institut de Biologie Intégrative et des Systèms (IBIS), Université Laval, 1030 Avenue de la Medecine, Québec, QC Canada

**Keywords:** Copepoda, Evolution, *Lepeophtheirus salmonis*, Reproduction, Sea lice, Sex-bias, Sexual dimorphism, Transcriptomics

## Abstract

**Background:**

Salmon lice, *Lepeophtheirus salmonis* (Copepoda: Caligidae), are highly important ectoparasites of farmed and wild salmonids, and cause multi-million dollar losses to the salmon aquaculture industry annually. Salmon lice display extensive sexual dimorphism in ontogeny, morphology, physiology, behavior, and more. Therefore, the identification of transcripts with differential expression between males and females (sex-biased transcripts) may help elucidate the relationship between sexual selection and sexually dimorphic characteristics.

**Results:**

Sex-biased transcripts were identified from transcriptome analyses of three *L. salmonis* populations, including both Atlantic and Pacific subspecies. A total of 35-43 % of all quality-filtered transcripts were sex-biased in *L. salmonis*, with male-biased transcripts exhibiting higher fold change than female-biased transcripts. For Gene Ontology and functional analyses, a consensus-based approach was used to identify concordantly differentially expressed sex-biased transcripts across the three populations. A total of 127 male-specific transcripts (i.e. those without detectable expression in any female) were identified, and were enriched with reproductive functions (e.g. seminal fluid and male accessory gland proteins). Other sex-biased transcripts involved in morphogenesis, feeding, energy generation, and sensory and immune system development and function were also identified. Interestingly, as observed in model systems, male-biased *L. salmonis* transcripts were more frequently without annotation compared to female-biased or unbiased transcripts, suggesting higher rates of sequence divergence in male-biased transcripts.

**Conclusions:**

Transcriptome differences between male and female *L. salmonis* described here provide key insights into the molecular mechanisms controlling sexual dimorphism in *L. salmonis*. This analysis offers targets for parasite control and provides a foundation for further analyses exploring critical topics such as the interaction between sex and drug resistance, sex-specific factors in host-parasite relationships, and reproductive roles within *L. salmonis*.

**Electronic supplementary material:**

The online version of this article (doi:10.1186/s12864-016-2835-7) contains supplementary material, which is available to authorized users.

## Background

Sexual dimorphism describes the phenotypic differences between sexes of the same species. It is ubiquitous across the animal kingdom and is favored through a combination of sexual selection, intersexual competition for resources, and fundamental differences in reproductive roles [1–3]. Genes overexpressed in one sex relative to the other are known as sex-biased genes, and include genes expressed in both sexes (but higher in one) or genes expressed in only one sex (sex-specific; reviewed in [4, 5]). A large proportion, often greater than 50 %, of genes exhibit sex-biased expression in many species including fruit flies *Drosophila* spp*.* [6, 7]*,* the nematode *Caenorhabitis elegans* [8]*,* parasitic flatworms *Schistosoma* spp. [9, 10]*,* the water flea *Daphnia pulex* [11]*,* the African clawed frog *Xenopus laevis* [12]*,* the songbirds *Taeniopygia guttata* and *Sylvia communis* [13], the olive flounder *Paralichthys olivaceus* [14]*,* the mouse *Mus musculus* [15], and humans *Homo sapiens* [16, 17]. This trend is largely driven by expression differences in the gonad. As such, transcriptome profiling is a highly useful approach to understand the mechanisms underlying sexual dimorphism and reproduction.

Crustaceans are one of the most diverse animal taxa, comprising more than 850 families with approximately 67,000 species [18, 19]. They are ecologically important, serving essential roles in the food chain and primary production in marine ecosystems [20]. Furthermore, crustaceans play important roles in aquaculture as both farmed animals (62 species worth over USD 34.8 billion per year; [21, 22]) and as parasites of farmed fish [23]. Most parasitic crustaceans are species within the class Copepoda, which displays a vast array of sexual dimorphism in anatomy, reproductive roles, sensory systems, and host/parasite relationships [23]. One of the most studied parasitic copepod, the salmon louse *Lepeophtheirus salmonis*, causes more than USD 480 million in losses to the Atlantic Salmon (*Salmo salar*) aquaculture industry annually [24]. Additionally, drug resistant strains of *L. salmonis* (and other sea lice species) have emerged globally (reviewed by [25]), threatening the sustainability and productivity of the industry.

*Lepeophtheirus salmonis* displays sexual dimorphism among several morphological, physiological, and behavioural characters. This phenomenon is observed in the motile parasitic stages of the lice (pre-adult I, II, and adult) although sex-specific differences in cephalothorax size and molt timing are also evident at preceding stages [26]. In addition, males develop faster than females, but they mature at approximately half the size of the adult female [26–28]. Sex differences in the morphology of the genital segment, abdomen, and appendages occur in all motile stages [27]. Distinct sex-associated behavioral characteristics related to reproductive success including frequency of host switching [29–31], mate location [32], blood feeding [33], and mate-guarding [31, 34] have also been reported. Sexually dimorphic physiology is also evident when *L. salmonis* are exposed to a commonly used antiparasitic compound, emamectin benzoate (EMB). Although EMB-resistance is widespread [25], males consistently show higher tolerance to EMB compared with females, regardless of the overall level of resistance within the population [35–37]. However, the molecular mechanisms underpinning sex-specific anatomy, behavior, and physiology in *L. salmonis*, and copepods in general, remain poorly understood.

The present study investigates sex-biased gene expression in three populations of *L. salmonis* using newly-generated transcriptomic data from Pacific Canada *L. salmonis* as well as a novel analysis of an available published dataset from Atlantic Canada *L. salmonis* [37]. A consensus-based, meta-analysis approach was used to identify sex-biased transcripts putatively responsible for sexual dimorphism in *L. salmonis*. Additionally, *L. salmonis* sequence conservation with related species (UniProt or Conserved Domain Database; e < 10^-10^) was integrated with sex-biased expression results to investigate sex-specific selective pressure and genomic constraint.

## Results

### Sex-biased gene expression in *L. salmonis*

**Fig. 1:**
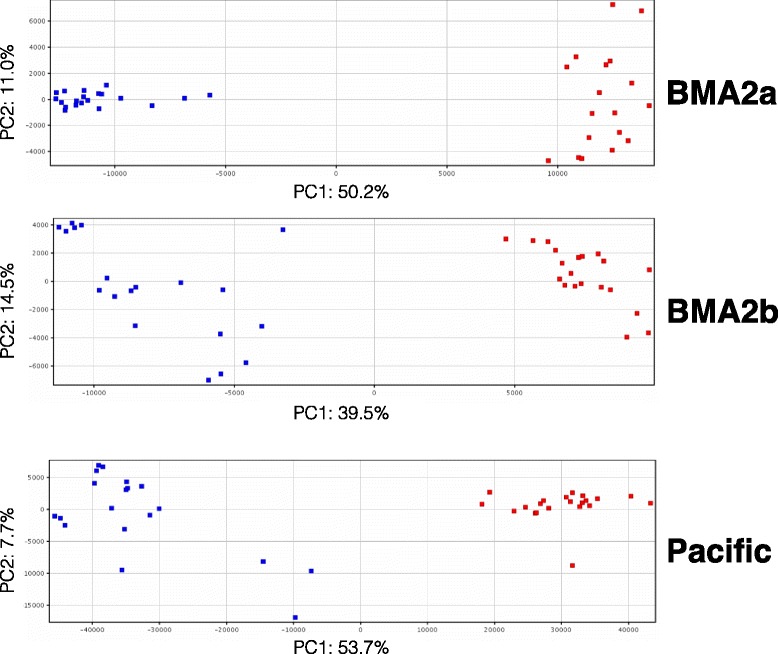
Principal Component Analysis of three populations of *L. salmonis.* Individual lice are represented in *blue* (males) and *red* (females). Sexes are separated on PC1 (x-axis) in all populations by 50.2 %, 39.5 % and 53.4 % for BMA-2a, BMA-2b, and Pacific lice, respectively. Only transcripts expressed by both sexes (i.e. excluding sex-specific probes) were included in PCA analysis

Sex-biased transcripts were identified in three populations of pre-adult II *L. salmonis* using a 38 K oligonucleotide microarray. Two of the populations were from the Atlantic subspecies *L. salmonis salmonis* and were collected from separate bay management areas (BMA-2a and BMA-2b) in the Bay of Fundy, New Brunswick [37], and the third was from the Pacific subspecies *L. salmonis oncorhynchi* [38] collected from the Broughton Archipelago, British Columbia (BC). Eighteen to 21 F1 generation preadult males and females from each population were analyzed in individual microarray hybridizations (total *n* = 117 individuals and hybridizations). A total of 34.7 – 42.7 % of all unique contigs passing quality control (QC) filters were significantly sex-biased (Benjamini-Hochberg multiple test correction; *p* < 0.01; fold change (FC) ≥ 1.5) in Atlantic and Pacific *L. salmonis* (Table 1). Including only the transcripts expressed in both sexes, a Principal Component Analysis (PCA) separated male and female samples along the first principal component (PC1; explaining the most variation) in all three populations, representing 50.2, 39.5 and 53.4 % of the transcriptional variation in BMA-2a, BMA-2b, and Pacific lice, respectively (Fig. 1). No consistent differences were observed between the proportions of transcripts overexpressed in males relative to females in each population (Table 1). Sex-biased transcripts for each population, including p-values, fold changes, annotations, and accession identifiers, can be found in Additional file 1.

**Table 1:** Sex-biased contigs in three populations of *L. salmonis*

*L. salmonis* populations	Unique contigs passing QC filter	Proportion sex-biased (%)	Male-biased contigs	Female-biased contigs	Proportion (%) of orphans		
					Male-biased	Female-biased	Unbiased
Atlantic (BMA-2a)	11859	34.7	1955	2157	45.1	37.1	33.1
Atlantic (BMA-2b)	8527	40.0	1729	1682	48.9	32.8	32.4
Pacific	14923	42.7	3068	3303	51.8	28.3	34.0
Consensus	7889	N/A	368	461	50.7	20.1	N/A

Orphans are contigs without annotation (BLASTx; e < 10^-10^). The numbers listed in this table are representative of unique contigs (i.e. duplicate probes removed). Only unique contigs were considered for each category

### Sequence conservation in sex-biased transcripts

The *L. salmonis* contigs used for microarray construction [39] were annotated using BLASTx and RPS-BLAST against SwissProt and Conserved Domain Database (CDD; [40]), respectively [39]. Contigs without annotation (e > 10^-10^) are marked as *unknown* in the additional files. The proportion of orphans relative to annotated transcripts in male-biased, female-biased, and unbiased categories was assessed for each population. This approach has been used in other model organisms, for example in flies and nematodes [11, 41]. Male-biased transcripts from all three *L. salmonis* populations had a higher proportion of orphans compared with female-biased and unbiased transcripts (Table 1). Female-biased and unbiased transcripts did not show consistent differences in the proportion of orphans (Table 1). These data suggest lower sequence conservation of male-biased transcripts in *L. salmonis*.

### Consensus of sex-biased transcripts in three populations of *L. salmonis*

**Fig. 2:**
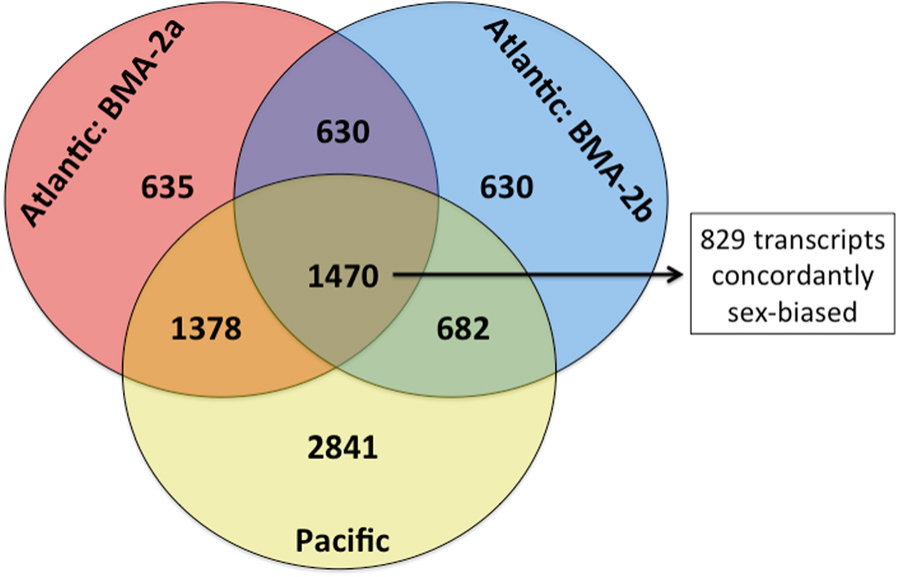
A consensus approach to identify sex-biased transcripts in *L. salmonis.* Sex-biased transcripts are displayed for each population separately. A consensus on sex-biased expression was achieved by creating a list of transcripts showing concordant differential expression between males and females across all three populations. Sex-biased transcripts for each individual population analysis can be found in Additional file 1 while consensus transcripts are displayed in Additional file 2

To assess the functional impacts of sex-biased expression in *L. salmonis*, differentially expressed transcripts between sexes from each population were used to generate a consensus list (Fig. 2). A total of 1470 unique transcripts, out of a total of 7889 were shown to be significantly sex-biased in all three populations with 829 of these showing concordant expression profiles (Fig. 2; Additional file 2). Using this consensus list (i.e. requiring concordant differential expression being identified in all three populations), 368 transcripts showed male-bias and 461 were female-biased. As expected from the individual population analyses, consensus male-biased transcripts showed a 2.5-fold higher proportion of orphans compared with those showing female-bias (Table 1). Differences in the degree of sex-biased expression, as measured by fold change (FC) also varied between male- and female-biased transcripts. On average, 84.8 % of the transcripts overexpressed in females had low sex-bias (FC ≥ 1.5 and ≤ 4), whereas transcripts overexpressed in males had equal proportions of high and low sex-bias (Table 2). Interestingly, 127 male-biased transcripts were not expressed above background levels in any of the 58 females assayed and therefore are referred to as male-specific. In contrast, only 20 transcripts were female-specific in the consensus list. Here, fold changes are reported as the range of differential expression between males and females across all populations, unless the transcript was sex-specific, and then it is denoted as such. Fold changes specific to each population for consensus sex-biased transcripts can be found in Additional file 2.

**Table 2:** Extent of sex-bias in three *L. salmonis* populations using consensus sex-biased transcripts (Additional file 2)

Sex-bias	Population	Proportion (%)	
		Low fold change (≥1.5 but < 4)	High fold change (≥4 fold)
MALE	BMA2a	51.5	48.5
	BMA2b	55.3	44.7
	Pacific	37.7	62.3
	Mean FC	49.9	50.1
FEMALE	BMA2a	85.5	14.5
	BMA2b	98.5	1.5
	Pacific	77.3	22.7
	Mean FC	84.8	15.2

Mean fold change (FC) was calculated for each transcript using the average fold change across the three populations

### Male-biased transcripts in three populations of *L. salmonis*

**Fig. 3:**
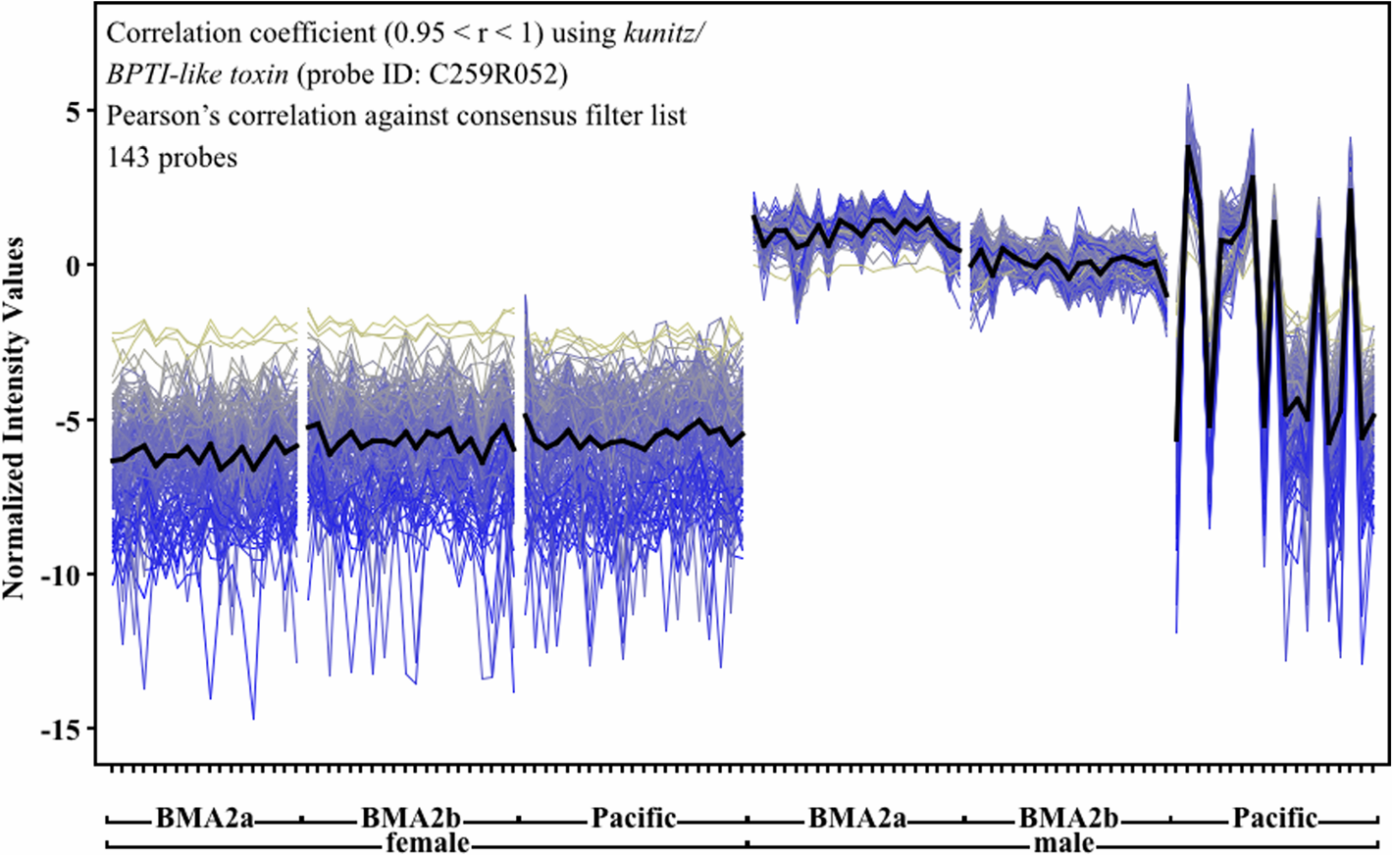
Co-expressed transcripts with putative roles in male reproduction. A total of 110 transcripts were co-expressed with *BPTI/kunitz-like toxin* (probe ID: C259R052) based on Pearson’s correlation (0.95 < r < 1) against the consensus QC-filtered transcript list (Table 1). Males and females are separated into each population on the x-axis. Normalized relative intensities (y-axis) are represented by log-2 Cy5/Cy3 ratios

The majority of annotated male-biased transcripts had roles in reproduction, for example being accessory gland proteins (Acps) and seminal fluid proteins (SFPs; reviewed by [42]). Transcripts known to regulate proteolysis for reproduction-related functions were highly male-biased in *L. salmonis* and included 16 proteases and 13 protease inhibitors, 10 of each being male-specific. However, a high degree of variance was observed in the expression of proteolytic transcripts among Pacific males (Fig. 3). To better understand this expression pattern, a transcript similarity assessment using *kunitz/BPTI-like toxin* (probe ID: C259R052) showed that 110 transcripts were strongly co-expressed (Pearson’s correlation, 0.95 < r < 1.0; Fig. 3). Although Atlantic males showed constitutive expression of these transcripts, Pacific males showed a characteristic “on/off” expression profile, with 10 of 19 individuals showing low, or absence of expression (Fig. 3). This co-expressed transcript list contains numerous representatives from known functional categories of male reproduction including peroxidases, pH regulators, kinases, and transporters, among others (Table 3). As seminal fluid proteins (SFPs) are only expressed in males [43], transcripts exhibiting male-specific expression are putatively assigned as candidate SFPs in *L. salmonis*. Many of these transcripts also enriched the Swiss-Prot (SP) and Protein Information Resource (PIR) Keyword (SP_PIR_Keyword) secreted (19 transcripts; *p* < 0.0001, Additional file 3), further supporting the involvement of these transcripts as SFPs or accessory gland proteins (reviewed by [42]).

**Table 3:** Transcripts linked to male reproduction based on expression profiles and annotation

Functional category	Transcript description	Degree of Male-bias (FC)	Probes	SwissProt Accession	CDD Accession	Ref
Protease inhibitors	Keratin-associated protein 6-2	30.8 - 277.1	C088R043	O08884	smart00131	[95, 96]
	Kunitz_BPTI	MS	C084R101	NA	smart00131	[69, 95–100]
	Kunitz_BPTI	MS	C057R056	NA	smart00131	
	Kunitz_BPTI	MS	C259R083	NA	pfam00014	
	Kunitz/BPTI-like toxin^a^	MS	C259R052	B5L5M7	pfam00014	
	Papilin^a^	MS	C063R028	Q868Z9	pfam00014	[101]
	Papilin^a^	MS	C183R014			
	Papilin^a^	MS	C066R049			
	Papilin^a^	MS	C142R005			
	Tissue factor pathway inhibitor 2^a^	MS	C213R048	O35536	pfam00014	[100, 102]
	Antichymotrypsin-2^a^	2.9 - 4.2	C077R006	P80034	cd00172	[59, 95, 96, 101, 103]
	SERine Proteinase INhibitors (serpins)	MS	C215R048	NA	cd00172	
	Serpin-Z10	1.6 - 2.4	C182R015	Q9SIR9	cd00172	
Kinases	Adenylate kinase isoenzyme 1	2.1 - 10.4	C104R155	P05081	TIGR01360	[96, 104]
	Adenylate kinase isoenzyme 1	1.9 – 4.8	C153R147	P00571	TIGR01360	
	Adenylate kinase isoenzyme 1	2.1 - 7.1	C031R080			
	Casein kinase I isoform alpha	MS	C070R100	P97633	cd14016	[96, 105]
	Casein kinase I isoform epsilon	1.8 - 4.8	C244R145	Q9JMK2	cd00180	
	Hexokinase type 2	2.5 - 7.9	C066R139	Q9NFT7	COG5026	[95, 106]
	Probable adenylate kinase isoenzyme F38B2.4	1.9 - 5.7	C212R032	Q20140	TIGR01360	[96, 104]
	Probable adenylate kinase isoenzyme F38B2.4	2.0 - 5.7	C028R063			
	Pyruvate kinase	2.3 – 5.0	C020R004	O62619	pfam00224	[95, 96]
	Pyruvate kinase	2.0 – 4.6	C015R041		cd00288	
	Pyruvate kinase	1.9 - 5.0	C155R159			
Proteases	Calpain-A catalytic subunit	MS	C197R005	Q11002	smart00720	[95, 102]
	Calpain-A catalytic subunit	MS	C018R134		smart00230	
	Carboxypeptidase B^a^	MS	C161R058	P04069	cd03860	[102, 106]
	Cytosolic non-specific dipeptidase	MS	C261R120	Q3ZC84	COG0624	[102, 106, 107]
	Cytosolic non-specific dipeptidase	1.9 - 2.9	C145R086	Q9D1A2	COG0624	
	Proprotein convertase subtilisin/kexin type 5^a^	2.5 - 4.2	C118R013	Q9NJ15	cd00064	[96]
	Serine protease persephone	MS	C118R020	Q9VWU1	smart00020	[61]
	Testisin	2.2 – 9.8	C007R130	Q9JHJ7	smart00020	[108, 109]
	Tryp_SPc, Trypsin-like serine protease	5.5 – 322.5	C158R134	NA	smart00020	[95, 96, 103]
	Tryp_SPc, Trypsin-like serine protease	MS	C009R051			
	Tryp_SPc, Trypsin-like serine protease	MS	C008R159			
	Zinc metalloproteinase nas-15^a^	2.1 - 3.7	C134R018	P55115	cd04280	[95, 103]
	Prostasin^ab^	MS	C135R082	Q16651	cd00190	[102, 110]
	ZnMc_adamalysin_II_like	MS	C083R024	NA	cd04269	[96, 102]
	Proclotting enzyme heavy chain	MS	C006R078	P21902	smart00020	[111]
	Gamma-glutamyltranspeptidase 1^b^	1.7 – 3.4	C120R152	P20735	cl19223	[112]
pH regulation	Carbonic anhydrase 1	MS	C183R004	P83299	cd00326	[96, 113–115]
	Carbonic anhydrase 9	MS	C196R116	Q8VHB5	cd00326	
	Carbonic anhydrase 9	MS	C131R016			
	Carbonic anhydrase 9	MS	C161R087			
Structural	Actin	MS	C223R146	Q92192	PTZ00004	[106, 116]
	Lamin Dm0	1.5 - 6.6	C220R106	P08928	pfam00038	[117]
	Outer dense fiber protein 2-like	2.5 - 5.2	C121R150	Q08B20	pfam02463	[51, 106]
	Kelch-like protein 20	MS	C022R130	Q5R7B8	NA	[96, 118]
	Tubulin alpha-2 chain	MS	C160R074	P06604	cd02186	[95, 106]
Transport	Solute carrier family 15 member 1	4.1 - 9.3	C170R033	P46059	TIGR00926	[96, 106]
	Solute carrier family 2, facilitated glucose transporter member 1	1.5 - 2.8	C192R047	P11166	pfam00083	
	Sodium/glucose cotransporter 4	1.6 – 3.1	C170R069	Q2M3M2	pfam00474	
	Solute carrier family 22 member 6-B	2.5 - 4.3	C072R016	Q66J52	TIGR00898	
	Sodium-dependent nutrient amino acid transporter 1	MS	C167R125	B4JMC1	pfam00209	
	Aquaporin-12A^b^ (*LsGlp1_v1*; [81])	1.7 - 5.3	C096R035	Q8IXF9	NA	[96, 119]
	Aquaporin-3 (*Lsaqp12L2*; [81])	3.7 - 10.4	C030R103	Q8R2N1	cd00333	
Other	Mucin-like glycoprotein	3.3 - 9.9	C218R155	NA	pfam01456	[102, 120]
	Major royal jelly protein 3^a^	MS	C089R070	Q17060	NA	[121–124]
	Chorion peroxidase heavy chain^a^	MS	C176R138	Q9VEG6	pfam03098	[125, 126]
	Chorion peroxidase heavy chain^a^	MS	C154R094			
	Peroxidase^a^	MS	C026R132	Q01603	pfam03098	
Energy	Fructose-bisphosphate aldolase	2.0 – 3.4	C230R040	O52402	PRK09197	[59, 80, 104]
	Fructose-bisphosphate aldolase	1.8 – 3.0	C085R145	P14540	PRK09197	
	Fructose-bisphosphate aldolase	1.9 – 3.3	C069R104	P14540	cd00946	
	Glycogen phosphorylase	1.7 – 2.5	C107R029	Q9XTL9	cd04300	
	Glycogen phosphorylase	1.6 – 3.2	C085R148	Q9XTL9	cd04300	
	Glycogen phosphorylase, brain form	1.6 – 3.5	C171R004	Q3B7M9	cd04300	
Male Fertility	Protein ref(2)Pb	1.6 - 3.9	C036R126	Q24629	cd14320	[127]

References are provided based on the identification of similarly annotated sequences or proteins involved in male reproduction (e.g. spermatogenesis, seminal fluid proteins, testis expression) in related organisms. The degree of male-bias is indicated by a range of fold change (FC) across populations while MS indicates the listed probes showed male-specific expression. Transcripts denoted with ^a^ have a signal peptide for secretion (SwissProt) while those with ^b^ were annotated using e < 10^-5^ due to the absence of homology at higher stringency. Transcripts without annotation are represented by NA (no annotation). Each transcript has a unique contig ID which can be found in Additional file 2 using the probes listed here

**Fig. 4:**
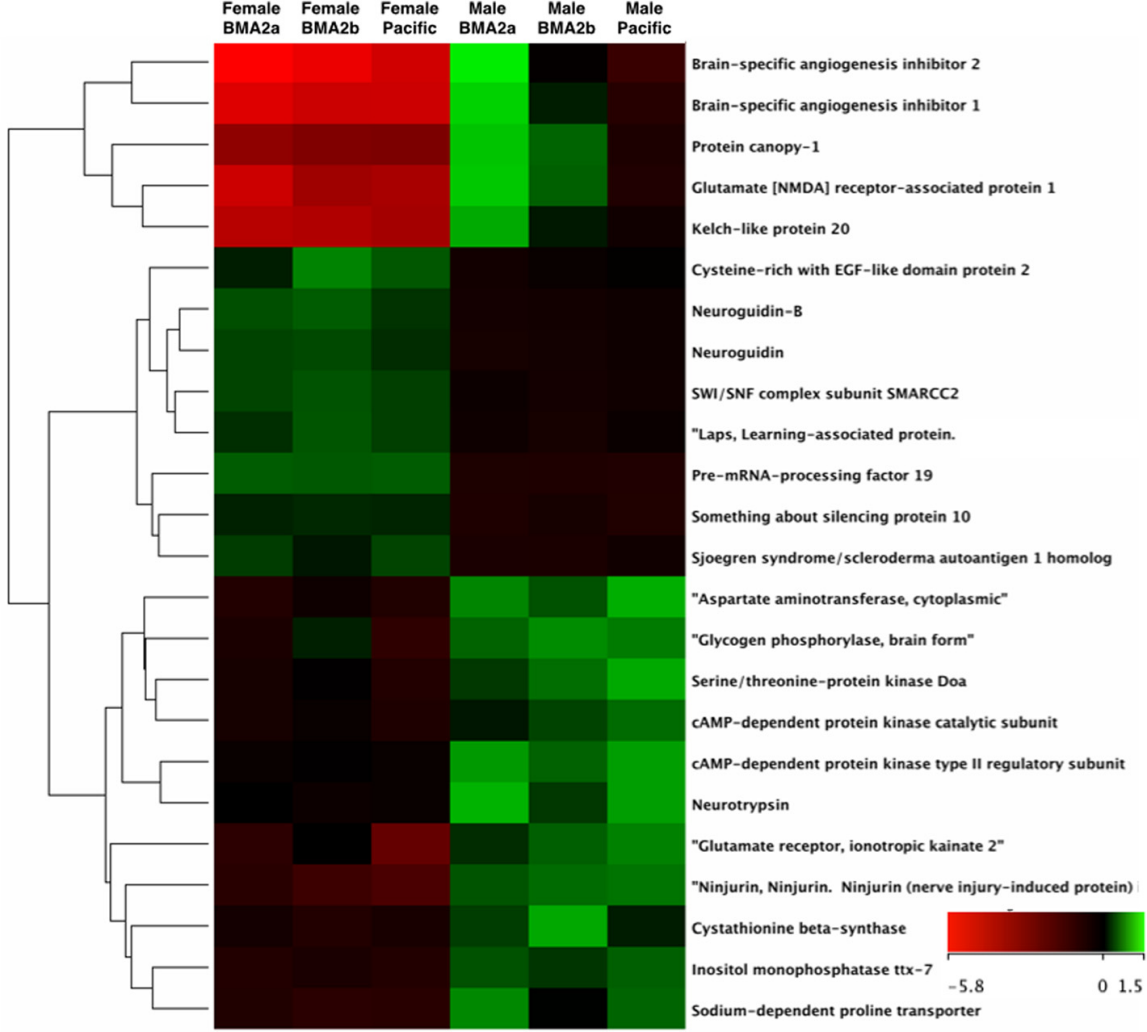
Candidate sex-biased transcripts involved with sensory system related functions in *L. salmonis.* Transcripts with high expression relative to Cy3 reference pool are *green* while low expressing transcripts relative to the Cy3 reference pool are *red*

Several other male-biased transcripts had putative roles in morphogenesis and the nervous system. Male-biased transcripts were enriched for cellular component assembly involved in morphogenesis (4 transcripts, *p* = 0.03), ossification (here probably calcification; 4 transcripts, *p* = 0.02), and Z disc (5 transcripts, *p* < 0.0001; Additional file 3). Additionally, male-biased transcripts were enriched for potassium ion binding (4 transcripts, *p* = 0.02), calcium ion binding (12 transcripts, *p* = 0.03), ion channel activity (5 transcripts, *p* = 0.04), and solute:cation symporter activity (5 transcripts, *p* = 0.01; Additional file 3), showing differences in sensory-system related functions. Other sex-biased transcripts involved in the nervous system, including their sex-biased expression profiles, are reported in Fig. 4. As salmon lice display sexually dimorphic patterns of mobility (i.e. mate location [32] and frequency of host switching [31]) and responses to neurotoxic drugs [37, 44], these transcripts will serve as important markers to better understand sex-related differences in the *L. salmonis* sensory system.

### Female-biased transcripts in three populations of *L. salmonis*

Female-biased transcripts were enriched for basic molecular processes including RNA processing (60 transcripts, *p* < 0.0001), ribosome biogenesis (38 transcripts, *p* < 0.0001), and transcription (41 transcripts, *p* < 0.0001) (Additional file 3). Within these categories, some multi-subunit protein complexes were completely female-biased including chaperonin-containing T-complex (CCT-complex; 5 transcripts, *p* = 0.0002), Nup107-160 complex (4 transcripts, *p* = 0.02), spliceosome (11 transcripts, *p* = 0.02), histone deacetylase complex (4 transcripts, *p* = 0.04) and ribonucleoprotein complex (30 transcripts, *p* = 0.0009). Protein complex formation was also enriched in the female-biased list, for example the GO category macromolecular complex subunit organization (28 transcripts, *p* < 0.0001).

Several transcripts related to cell division and organization were overexpressed in females and some significantly enriched GO categories included cell cycle (35 transcripts, *p* < 0.0001), DNA replication (17 transcripts, *p* < 0.0001), and cell proliferation (13 transcripts, *p* = 0.004) (Additional file 3). Furthermore, GO enrichment of ATP binding (60 transcripts, *p* < 0.0001), ATP-dependent helicase activity (10 transcripts, *p* = 0.001), and ATPase activity (15 transcripts, *p* = 0.005; Additional file 3) indicated female-biased energy generation.

As observed with male-biased transcripts, female-biased transcripts were also enriched for reproductive functions. The GO category *in utero* embryonic development (8 transcripts, *p* = 0.005; Additional file 3) was significantly enriched despite the absence of mating across all experiments (female lice are not sexually mature at the pre-adult stage; [34]). These included *nuclear autoantigenic sperm protein* (FC = 2.7 – 10.0), *pre-mRNA processing factor 19* (FC = 1.6 – 2.6), and *protein arginine N-methyltransferase 1*, among others (FC = 1.7 – 2.5; Additional file 3). Female-biased transcripts were also enriched for nuclear hormone receptor binding (4 transcripts, *p* = 0.02) with an additional female-specific transcript containing the c4 zinc finger common to nuclear hormone receptors (*Zn_C4*; CDD: smart00390; e = 2.14^-15^). Several other female-biased transcripts not included in GO categories also have potential roles in reproduction including *piwi-like protein 1* (FC = 2.6 – 8.3) and *peroxiredoxin 1* (FC = 1.6 – 3.1). Lastly, transcripts involved in sex determination were female-biased in all populations, including *prohibitin-2* ([45]; FC = 1.5 – 9.6) and *pre-mRNA-splicing regulator female-lethal(2)D* ([46]; FC = 1.7 – 2.4).

Some transcripts related to morphology, feeding, and detoxification were also female-biased across all populations. Transcripts such as *serine proteinase stubble catalytic chain* (female-specific), *la protein homolog* (FC = 1.5 – 2.5), and *digestive organ expansion factor homolog* (FC = 1.7 – 2.9), are involved in development, while *trypsin-1* (FC = 3.1 – 12.0) and *quinone oxidoreductase* (FC = 2.1 – 116.5) have putative roles in feeding and detoxification, respectively. Lastly, immune-related transcripts, like *rhotekin-2* (female-specific), *ras-related protein Rab-32* (female-specific), and *complement component 1 Q subcomponent-binding protein, mitochondrial* (FC = 1.6 – 4.0), were female-biased in all *L. salmonis* populations. Female-biased transcripts therefore represent several candidates responsible for controlling sexual dimorphism at the molecular level in *L. salmonis*. Based on these findings, immunity, energy expenditure, and organogenesis are examples of previously unknown sexual dimorphism in salmon lice.

### Annotation of *L. salmonis* sex-biased orphan contigs

Sex-biased orphan contigs were compared to known sequences using UniProt (e ≤ 10^-5^) to augment novel transcript discovery in the non-model *L. salmonis*. Although this threshold is less conservative than that used for the original microarray annotation [39], it has been frequently used in other sea lice transcriptomic studies [47–49], being generally acceptable for gene annotation [50]. This method was used for novel transcript discovery only and these newly annotated transcripts were not included in GO analyses. A total of 16 female-biased and 12 male-biased transcripts were subsequently annotated (Additional file 2) using this method. This additional annotation did not substantially change observations made on sequence divergence differences in male and female-biased transcripts, and many of the newly annotated female-biased transcripts had similar functions to those identified in the enrichment analysis described above, including transcription, translation, and cell cycle (Additional file 3).

Potential links to reproduction were observed among several of the newly annotated male-biased orphans, including *prostasin* (male-specific), *gamma-glutamyltranspeptidase* (FC = 1.7 – 3.4), and *protein ref(2)P* (FC = 1.6 – 2.3) (Table 3). A male-specific transcript, *c-factor*, was also identified. However, the function of this transcript in *L. salmonis* remains unknown. Additionally, male-biased transcripts involved in neuromuscular development and function were discovered, including *excitatory amino acid transporter 3* (*SLC1A1*; FC = 2.3 - 3), *twitchin* (FC = 1.6 – 5.7), and *sarcoplasmic/endoplasmic reticulum calcium ATPase 1* (FC = 1.5 – 7.6). Probe identifiers, e-values, bitscores, SwissProt accessions, and descriptions for newly annotated transcripts can be found in Additional file 2.

## Discussion

The economically and ecologically important parasite, salmon lice *L. salmonis* (Copepoda: Caligidae) displays sexual dimorphism in ontogeny, morphology, physiology, and behavior [26–35]. However, little is known about the molecular mechanisms that control these traits and the possible interactions they may have with chemical response/resistance, host-parasite interactions, and overall population dynamics. Here, sex-biased transcripts were identified within three populations of *L. salmonis* (including two subspecies; [38]) from the Atlantic and Pacific coasts of Canada. Our observation revealed a large proportion (34.7 – 42.7 %) of sex-biased expression (Table 1) consistent with findings in other arthropods [6, 11], nematodes [8], amphibians [12], fish [51], birds [13], and mammals [17]. Principal component analysis supported this finding as males and females were separated on PC1 in all populations. These data suggest large differences in baseline gene expression between male and female *L. salmonis* that could potentially impact parasite control strategies. For example, routine lice counting and staging is the primary management strategy used to inform treatment regimes in aquaculture. However, pre-adult male and female *L. salmonis* are often grouped into a single category for these counts [52, 53]. The basal differences in gene expression reported here and the interactive effects of sex with chemical treatment and resistance described elsewhere [37], suggests a more informative strategy would be to separately count male and female preadult *L. salmonis* in both farm and laboratory settings whenever possible.

There were no consistent differences between the proportion of male-biased relative to female-biased transcripts in *L. salmonis* (Table 1), despite observations in other crustaceans like *Caligus rogercresseyi*, another species of parasitic copepod, and *Daphnia pulex* which suggested a slight over-representation of male-biased transcripts [11, 54]. However, we did find that a larger proportion of male-biased transcripts had higher fold change than those with female-bias. In this study, sex-biased expression was assessed across multiple populations of *L. salmonis* reared under similar conditions, thus offering a more comprehensive overview of gene expression differences than is obtained in single cohort transcriptome analyses. However the sex-biased transcripts identified here were limited to ~22,000 unique contig sequences (i.e. transcripts) on the microarray and it was not possible to examine some of the more complex facets of sex-biased expression (i.e. sex-specific alternative transcripts [55]). Additionally, studies using whole-body homogenates offer general patterns of sex-bias but lack the resolution to detect sex-biased expression in individual tissues [7] and control for differences in tissue allometry [56]. Tissue-specific extractions will be an important tool for future studies examining sex-biased gene expression in *L. salmonis*. Nonetheless, characterization of sex-biased transcripts reported here provides numerous molecular targets putatively underlying sexual dimorphism in *L. salmonis*, offering important insight for pest management and future drug development for this non-model organism.

### Discovery of sex-biased transcripts related to reproduction in *L. salmonis*

Male-biased transcripts displayed greater sex-bias compared with female-biased transcripts in *L. salmonis*, similar to findings in *D. melanogaster* [6]. Among the highly male-biased transcripts, 127 were not expressed above background detection in any of the 58 females assayed in this study. These transcripts represent putative seminal fluid proteins (SFPs) based on their expression profiles (SFPs are only expressed in males; [43]) and annotations (Table 3 and references therein). SFPs are transferred to females during mating, impacting a variety of physiological processes such as sperm storage, egg production, feeding, behavior, and receptivity to re-mating (reviewed in [42]). Although genes involved in reproduction tend to evolve rapidly [57], many of the functional constituents of seminal fluid are conserved from arthropods to mammals [43, 58]. For example, genes involved in proteolysis are essential for sperm transfer, storage, and activity, among other functions [59, 60]. A total of 10 proteases and 10 protease-inhibitors were identified as male-specific in this study with several others showing male-bias (Table 3). All protease inhibitors putatively involved in reproduction contained at least one kunitz or serpin domain (CDD accessions: pfam00014 and cd00172, respectively), which are the predominant classes of protease inhibitors in seminal fluid [59]. In turn, proteases such as *serine protease persephone* and c*alpain-A catalytic subunit* also had relevant annotations to SFPs based on their ability to modulate *toll* signaling in *D. melanogaster* [61, 62]. The transfer of immunomodulatory and antimicrobial SFPs may aid females in defending against infections that are introduced during mating [60, 63]. Many of the *L. salmonis* proteases assigned as SFPs also contained trypsin-like domains (CDD: cd00190 and smart00020) including *prostasin, proclotting enzyme heavy chain*, and three additional transcripts without SwissProt annotation (i.e. CDD only). These transcripts represent important targets for understanding the proteolytic events controlling reproduction and mating in *L. salmonis*.

Female-biased transcripts also had putative reproductive functions, including five with nuclear hormone receptor activity, one of which was female-specific. Recently, a sex-linked SNP in the *L. salmonis prohibitin-2* gene with a pattern of female-biased expression was identified in three strains of *L. salmonis* from Scotland [45]. Similarly, a transcript annotated as *prohibitin-2* was female-biased in all three populations of *L. salmonis* assayed in this study. The *prohibitin*-2 gene is likely involved in sex determination [45] along with *pre-mRNA-splicing regulator female-lethal(2)D* [46], which was also female-biased in *L. salmonis*.

Overall, functional enrichment for reproduction was less clear in female-biased transcripts compared with male-biased transcripts. Many of the putative female-biased transcripts potentially involved with reproduction had GO annotation with many other functional categories and only a small number were female-specific. For example, *nuclear autoantigenic sperm protein* (*NASP*) and *histone deacetylase* 1 (*HD1*), were female-biased in *L. salmonis* and have reproductive roles in similar species [54, 64, 65]. However, these transcripts were in the enriched GO category *in utero* embryonic development (*p* = 0.005), which is taxonomically constrained [66] and inconsistent with the virgin status of the lice used here [34]. Furthermore, the functions of *NASP* and *HD1* differ in other species such as *C. elegans* in which they are important for female development and male-specific gene repression [67]. These discrepancies make it difficult to identify putative female-biased reproduction genes in the present work. In an earlier study by Eichner and colleagues [47], *L. salmonis* genes involved in reproduction were overexpressed in adult females compared to preadult II females. As the present study used preadult II females, it may have missed the identification of some female reproductive genes that are induced later in development than the stages evaluated here. Future studies assessing the expression and localization of these transcripts will offer insight on their exact functions. Much work remains to identify female-biased reproductive genes in *L. salmonis*, including the extensive changes likely to occur in female mating-responsive genes post transfer of male spermatophores (e.g. see [68, 71, 72]).

In general, sex-biased genes evolve faster than unbiased genes, with those exhibiting male-bias showing the highest rates of evolution (reviewed by [4]). This trend is heavily influenced by sex-biased transcripts involved in reproduction as these typically exhibit higher than normal rates of positive selection (reviewed by [4, 57]). Only male-biased transcripts showed a consistently higher number of orphans in each *L. salmonis* population (45.1 – 51.8 %) when compared to female-biased (28.3 – 37.1 %) and unbiased (32.4 – 34.0 %) transcripts. This trend was largely driven by the putative SFPs in this study (i.e. male-specific transcripts), of which 65.4 % were orphans. As higher rates of nucleotide substitutions are often caused by a relaxed functional constraint or greater positive selection on certain transcripts (reviewed in [4, 57]), higher-resolution analyses including non-synonymous/synonymous mutation ratios (*dN/dS*; [70, 71] and codon-usage bias [72, 73] will be important in clarifying the effects of selection on these *L. salmonis* genes. These analyses will yield important information regarding evolutionary processes affecting reproduction, population dynamics, and drug resistance. In particular, it will be important to identify whether genes involved in drug resistance are similar to those involved in sex-biased expression as this will provide insight on molecular mechanisms behind the higher rate of drug resistance in male *L. salmonis*. Specifically this will inform on whether the increased resistance is due to inherent physiological factors that differ between the sexes, or to the evolutionary rate of resistance mechanisms.

### Sex-biased transcription related to sexually dimorphic phenotypes of *L. salmonis*

*L. salmonis* exhibits extensive sexual dimorphism in morphology at the pre-adult and adult stages [27]. Several sex-biased transcripts discovered here have related functions including the male-biased transcripts enriching the GO categories cellular components involved in morphogenesis (4 transcripts, *p* = 0.03) and Z-disc (sarcomere; 5 transcripts, *p* < 0.0001). Additionally, *serine proteinase stubble catalytic chain* was female-specific in *L. salmonis* and is required for proper formation of appendages in *Drosophila* spp. [74]. Therefore, these transcripts represent targets for understanding sexually dimorphic morphology including features that are important in mating (described in [27, 31, 34]). The sex-biased orphans described here are also ideal candidates for understanding mate guarding, a common mating behavior in crustaceans, which is known to be sexually antagonistic [75]. Therefore, transcripts involved in mate guarding are unlikely to be annotated due to rapid sexual selection [75] and taxonomic constraint [50].

Other molecular links to sexual dimorphism in *L. salmonis* were observed in transcripts such as *longitudinal lacking protein*, *trypsin-1*, and *digestive organ expansion factor*, which were female-biased in this study and have roles in salivary gland, trachea, and digestive organ development and function [76, 77]. Based on female *L. salmonis* having a greater requirement for blood in the meal [33], these transcripts will serve as important markers for understanding sexually dimorphic feeding patterns and related host-parasite interactions.

Male-biased *L. salmonis* transcripts also enriched several GO categories related to the sensory system, which is generally known to be more refined in male copepods [23]. These included potassium ion binding (4 transcripts, *p* = 0.02), solute:cation symporter activity (5 transcripts, *p* = 0.01), and calcium ion binding (12 transcripts, *p* = 0.03), among others (Additional file 3). Males are known to transfer between salmonid hosts more frequently than females [30, 31] with mate location primarily being the responsibility of the male [32]. Thus, transcripts involved in the sensory system and muscle development (e.g. Z disc; Additional file 3) represent putative targets for understanding neuromuscular differences related to increased mobility in males. Furthermore, chemical cues are essential components of host and mate location in *L. salmonis* and serve to optimize the probability of mating through balancing the proportion of males relative to females on each host [29]. Chemosensory signaling and behavioral responses to non-host semiochemical treatments known to interfere with host recognition are sexually dimorphic in *L. salmonis* [32, 78]. Based on the high evolutionary rates of genes involved in chemoreception [79], sex-biased orphans should be considered candidate targets for understanding the chemical ecology of *L. salmonis*.

The identification of sex-biased transcripts in *L. salmonis* also provided preliminary evidence for sexually dimorphic characteristics previously unknown in salmon lice. For example, immune-related transcripts such as *rhotekin-2* and *ras-related protein Rab-32* were female-specific in this study while other related transcripts were female-biased (Additional file 2). Gene Ontology analysis also indicated a higher energy expenditure in female *L. salmonis* based on the female-biased expression of 15 transcripts enriching ATPase activity coupled and 60 transcripts enriching ATP binding (Additional file 3). This is a similar finding to the GO analysis of sex-biased expression in *D. pulex* [11], and may serve as an explanation for the lower frequency of inter-host transfer in female *L. salmonis* [30, 31]. Although male-biased transcripts such as *glycogen phosphorylase, fructose-bisphosphate aldolase*, and *hexokinase-2* enriched the GO category glucose metabolic process (7 transcripts; *p* =0.02), these are likely acting as an energy source for spermatogenesis [80]. These data indicate an increased basal energy demand in female *L. salmonis* that is important for understanding observed sex differences in drug tolerance and resistance [35, 37]. Collectively, the candidate sex-biased transcripts described here represent putative markers controlling energy expenditure, morphology, and the immune and sensory systems of *L. salmonis*.

### Strengths and limitations of using a consensus-based approach to identify sex-bias

Recently, seven aquaporin paralogs were characterized in *L. salmonis*, each with different expression profiles across stage and sex [81]. Two of these aquaporins (*LsGlp1_v1* (KR005660.1) and *Lsaqp12L2* (KR005666.1)) showed male-specific and male-biased expression, respectively. In the present work, two male-biased contigs annotated as *aquaporin 3* (BT121448.1) and *aquaporin-12A* (BT121051.1; see Table 3) showed more than 99 % sequence similarity to *LsGlp1_v1* and *Lsaqp12L2*, respectively (Additional file 4). Therefore, *aquaporin 3* and *LsGlp1_v1* appear to be the same transcript based on sequence alignment, as are *aquaporin-12A* and *Lsaqp12L2*. Here, *aquaporin 3* was only expressed above background fluorescence in one of the 58 females assayed, supporting the expression profile previously reported [81]. Additionally, *aquaporin-3* was shown to be highly male-biased in the closely related *C. rogercresseyi* [54], suggesting this gene is important for a male-specific function in salmon lice. This type of transcriptomic consensus will be important for functional categorizations in future sea lice studies.

Within the 1407 transcripts shown to be sex-biased in Atlantic and Pacific populations, only 829 showed concordant expression profiles. The majority of transcripts in the discordant list (Additional file 2) were involved in cuticle formation and molting and, therefore, transcripts that potentially oscillate in expression levels at different molt intervals [47, 82] were eliminated from this interpretation. However, the consensus identification of sex-biased transcripts in *L. salmonis* did prove to be over-conservative in some cases, causing particular transcripts with putative sex-bias to be overlooked. For instance, *trypsin-4* (probe: C054R168), annotated from *Anopheles gambiae* and involved in host seeking behavior and blood feeding [83], was female-specific in BMA2a and in Pacific *L. salmonis*. However, this probe did not pass quality filters in the BMA2b population and was eliminated from the consensus list. Female *L. salmonis* are known to feed more heavily on blood than males [33], with certain trypsins known to be involved in digestion and immune evasion on salmonid hosts [84–86]. Additionally, a similar transcript annotated to *trypsin-1* was female-biased in this study (Additional file 2). Therefore, monitoring individual population analyses from this work is also important for identifying potential sex-biased markers in *L. salmonis*. Nonetheless, the consensus set of sex-biased transcripts identified here supports the characterization of *L. salmonis* transcript as markers for reproduction, morphogenesis, behavior, and other sexually dimorphic traits for targeted approaches (i.e. knock-out/knock-down, recombinant production, *in vitro* characterization, etc.) in future studies. This improved understanding of sex-biased gene expression in *L. salmonis* will inform future studies examining host-parasite interactions, drug resistance, reproduction, and novel drug discovery.

## Conclusions

A consensus-based, meta-analysis approach was used to analyze the *L. salmonis* transcriptome, clearly identifying sex-biased transcripts associated with sexually dimorphic traits. Specifically, male-biased transcripts showed higher degrees of sex-bias and lower sequence similarity compared with female-biased transcripts. The enrichment of male-biased transcripts associated with reproduction was likely responsible for these trends. Our results provided insights into known and novel forms of sexual dimorphism in *L. salmonis* including immunity, energy expenditure, morphology, feeding, and mobility. These sexual dimorphisms will be important to consider for industry-relevant applications in areas such as parasiticidal drug response, reproductive roles, and host-parasite relationships. The current work shows that sex-biased gene expression is abundant in the pre-adult *L. salmonis* transcriptome and is likely to control several aspects of sexual dimorphism in this species.

## Methods

### *Lepeophtheirus salmonis* populations and collections

Adult *L. salmonis* were collected from Atlantic salmon aquaculture farms on the Atlantic and Pacific coasts of Canada. Two populations of Atlantic *L. salmonis* were collected in the spring of 2013 from Bay Management Area 2a (BMA-2a; Back Bay) and 2b (BMA-2b; Grand Manan) in the Bay of Fundy, New Brunswick (NB), as described in full previously [35, 37]. A third population representing Pacific *L. salmonis* was collected from the Broughton Archipelago, British Columbia (BC) in 2010. Atlantic and Pacific *L. salmonis* are considered allopatric subspecies [38]. For all collections, egg strings were removed from adult females and larvae reared to the infective copepodid stage in static seawater hatch systems as previously described [35]. Copepodids (F1 generation) were then used to infect Atlantic salmon (*Salmo salar*) and allowed to develop to the pre-adult II stage. Pre-adult (F1) lice from all populations were used in 24 h in vitro EMB bioassays (described below) before collection and storage at -80 °C for RNA extraction.

### Atlantic and Pacific *L. salmonis* Microarray Datasets

Two microarray datasets were used to compare sex differences in *L. salmonis* from the Atlantic (2013 collection) and Pacific (2010 collection) coasts of Canada. The Atlantic dataset was accessed from NCBI through Gene Expression Omnibus (GEO) accession GSE56024 [37]. In this study a total of 77 pre-adult Atlantic *L. salmonis*, 38 females and 39 males from BMA-2a and BMA-2b were exposed to four concentrations of EMB (0.1, 25, 300, and 1000 ppb) and a seawater control, as previously reported. The bioassay protocol was identical for both populations. This study compared the effects of EMB on *L. salmonis* including the interactions between population (BMA-2a is more EMB-resistant than BMA-2b; [37, 87]) and sex. However, baseline differences between males and females were not reported. The Pacific dataset was provided by the same laboratory group (B. Koop and S. Jones, *unpublished data*), which exposed 39 pre-adult Pacific *L. salmonis,* 21 females and 19 males, to low doses of EMB (0.01, and 0.1 ppb) or a seawater control. Lower doses of EMB were selected based on the high EMB-sensitivity of this population [88]. For all F1 generation cultures, lice were maintained in filtered sea water at 10 ± 2 °C and 32 ± 2 ppt. The Pacific dataset has been uploaded to GEO under the accession GSE73734.

A 38 K oligonucleotide microarray (eArray, Agilent) designed with expressed sequence tags (ESTs) from Atlantic and Pacific *L. salmonis* [39] was used to analyze all lice in this study. Annotation of each contig was completed using BLASTx and RPS-BLAST against SwissProt and Conserved Domain Database (e < 10^-10^), respectively. A total of 18 – 21 hybridizations for each sex and population combination (117 total hybridizations) were completed. Hybridizations were completed using methods for sample preparation, microarray hybridization, and scanning as previously reported [37, 89]. Briefly, all slides were scanned using a Perkin Elmer ScanArray® at 5 μm resolution and optimized PMT intensities (1-2 % of array spots saturated). Filtering and quantification was completed using Imagene 8.1 (Biodiscovery) before completing statistical analyses in GeneSpring GX v12.6 (Agilent). A quality control (QC) filtered probe list was created for each population (Table 1) with probes included for statistical analysis only if at least 65 % of the samples in any one condition had raw fluorescent intensities ≥ 500 and showed no poor quality spots.

### Sex-biased Gene Expression in *L. salmonis*

Microarray data was used to characterize baseline expression differences between male and female *L. salmonis*. Sex-biased probes were identified using a two-way ANOVA with sex and EMB as explanatory variables (Benjamini-Hochberg multiple test correction; *p* < 0.01; fold change (FC) ≥ 1.5). The effects of EMB on Atlantic *L. salmonis* transcriptomes was minor [37] and a significant transcriptomic effect was not detected in Pacific *L. salmonis* used here (i.e. no probes showed differential expression by EMB). The effects of EMB exposures were also controlled for in the statistical model by only using transcripts affected by a main effect of sex and through consensus-based analyses (described below). All probes with a main effect of sex for each individual population are compiled into Additional file 1.

Sex-biased probes from each population were used to create a consensus list for functional analyses. This list was limited to probes exhibiting significant and concordant sex-bias in all three populations of *L. salmonis* described here (Fig. 2). Sex-biased probes from individual population analysis can be found in Additional file 1, while sex-biased probes identified using the consensus sex-biased method can be found in Additional file 2. As duplicate probes represent unique contigs (i.e. transcripts) on the array [39], contig IDs are also included in the additional files. The variation between duplicate probes of consensus sex-biased transcripts is quantified in Additional file 2. Only unique transcripts are used to calculate the proportion of sex-biased expression in Atlantic and Pacific *L. salmonis*.

Differences in the degree of sex-bias were assessed by binning transcripts based on their degree of differential expression between male and female *L. salmonis* [6]. Transcripts with low sex-bias were those overexpressed by a fold change (FC) of ≥ 1.5 but < 4, while highly sex-biased transcripts had a FC ≥ 4. A mean FC value representing all three populations was also included (Additional file 2). Any transcript that did not pass the background QC filter in 100 % of the individuals within one sex was considered to be sex-specific in this study. However, based on lower limits of detection for microarrays, these transcripts may not be biologically sex-specific. A transcript similarity assessment was also completed using all QC-filtered probes against *kunitz/BPTI-like toxin* (probe ID: C259R052). Transcripts with similar expression patterns to *kunitz/BPTI-like toxin* were determined using a Pearson’s correlation (0.95 < r < 1.0). These transcripts are described in Additional file 2.

Functional enrichment of the consensus sex-biased transcript list was done using Gene Ontology (GO), InterPro, and SwissProt (SP) and Protein Information Resource (PIR) Keywords (SP_PIR_Keywords) with DAVID bioinformatics [90–92] using a modified Fisher’s exact test (*p* < 0.05; genes/enrichment category ≥ 4). Unique SwissProt accession ID’s were compared against a QC filter background list designed to include transcripts passing QC filters in all populations (Table 1). To reduce redundancy of Gene Ontology categories, GO Trimming was used with an 80 % soft trim threshold [93]. All GO analyses can be found in Additional file 3.

### Sequence conservation in sex-biased and unbiased transcripts

To investigate rates of sequence divergence in sex-biased transcripts, unique contigs passing QC filter for each population were binned into one of three categories: Male-biased (overexpressed by males), female-biased (overexpressed by females) or unbiased (no expression difference between sexes). All contigs on the microarray were annotated using UniProt [94] and Conserved Domain Database (NCBI, [40]) with the best match being the alignment with the lowest Expect value followed by the highest bitscore [39]. Transcripts with no significant match (e > 1.0^-10^) were considered orphans (labeled as “unknown” in additional files). To assess the degree of sequence conservation between male-biased, female-biased and unbiased transcripts, the proportion of orphans relative to annotated transcripts in each of these categories was assessed based on similar methodologies [11, 41]. Sequence conservation in male-biased and female-biased transcripts from the consensus list was also analyzed (Table 1).

### Novel transcript discovery using sex-biased orphans

Based on the robust approach using a consensus sex-biased transcript list, orphans showing concordant sex-bias across all populations were re-annotated using a less conservative threshold of e > 1.0^-5^. This annotation threshold is common amongst other sea lice transcriptomic studies and is generally considered an acceptable cut-off for annotation [50]. This annotation was used for exploratory transcript prediction but not for the main analysis or the Gene Ontology enrichment analysis. Sex-biased orphan annotation results can also be found in Additional file 2.

## Abbreviations

BMA, Bay Management Area; CDD, Conserved Domain Database; EMB, emamectin benzoate; FC, fold change; GEO, Gene Expression Omnibus; GO, Gene Ontology; PCA, principal component analysis; QC, quality filtered; SFP, seminal fluid proteins; SP_PIR_Keywords, SwissProt and Protein Information Resource Keywords.

## Additional files

Additional file 1: Sex-biased and sex-specific transcripts in individual populations. (XLSX 1955 kb) Additional file 2: Consensus sex-biased and sex-specific transcripts, co-expressed “kunitz cluster”, and newly annotated sex-biased orphans. (XLSX 340 kb) Additional file 3: ene Ontology, SP-PIR_Keywords, and InterPro. (XLSX 93 kb) Additional file 4: Sequence alignment of *L. salmonis* aquaporins. (TXT 16 kb)
